# Efficacy and safety of once-weekly basal insulin analogs versus daily basal insulin analogs in adults with type 2 diabetes: a systematic review and meta-analysis

**DOI:** 10.1007/s11892-026-01628-3

**Published:** 2026-06-12

**Authors:** Dimitrios Raptis, Sotirios Chiotis, Theodoros Papamichalis, Eleni Bekiari, Apostolos Tsapas, Thomas Karagiannis

**Affiliations:** 1 Albert Einstein College of Medicine, NYC Health & Hospitals, Jacobi/North Central Bronx, Bronx, NY USA; 2 Third Department of Cardiology, ‘Hippokration’ General Hospital, Aristotle University of Thessaloniki, Thessaloniki, Greece; 3 Gastroenterology Department, 251 Hellenic Air Force General Hospital, Athens, Greece; 4 Clinical Research and Evidence-Based Medicine Unit, Second Medical Department, Aristotle University of Thessaloniki, Thessaloniki, Greece

**Keywords:** Once-weekly insulin, Insulin efsitora, Insulin icodec, Type 2 diabetes mellitus

## Abstract

**Purpose of Review:**

Recently developed once-weekly basal insulin analogs, including insulin icodec and insulin efsitora alpha (efsitora), aim to improve treatment adherence and patient convenience compared with daily basal insulin. This systematic review and meta-analysis evaluated the efficacy and safety of once-weekly basal insulin analogs compared with daily basal insulin analogs in adults with type 2 diabetes mellitus (T2DM) and included 14 randomized controlled trials (RCTs), comprising 8,487 subjects.

**Recent Findings:**

Evidence supports that once-weekly basal insulin analogs achieve comparable glycemic control to daily basal insulin analogs in adults with T2DM, offering greater reductions in HbA1c and improvements in time in range, with a safety profile similar to that of daily insulin regimens. In our analysis, once-weekly basal insulin resulted in a greater reduction in HbA1c compared with daily basal insulin (mean difference [MD] -0.09%, 95% CI -0.15 to -0.03), greater improvements in time in range (TIR) (MD 1.86%, 95% CI 0.73 to 2.98) and higher odds of attaining an HbA1c < 7.0% (OR 1.32, 95% CI 1.07 to 1.63, I2 64.9%). Rates of level 2 or 3 hypoglycemia did not differ significantly.

**Summary:**

Once-weekly basal insulin provides glycemic control comparable to daily basal insulin, with modest improvements in glycemic outcomes and similar hypoglycemia risk. Its primary advantage lies in treatment simplification and reduced injection burden, supporting its role as a convenient alternative to daily basal insulin in adults with T2DM.

**Supplementary Information:**

The online version contains supplementary material available at 10.1007/s11892-026-01628-3.

## Introduction

Despite advances in treatment of type 2 diabetes mellitus (T2DM), a significant proportion of people with T2DM struggle to maintain optimal glycemic control. Management of T2DM is multifaceted beginning with lifestyle modifications, while pharmacologic therapy is individualized based on comorbidities, risk of hypoglycemia, and patient preferences. Insulin therapy is indicated when glycemic control is not achieved with oral and/or non-insulin injectable agents, or when there is symptomatic hyperglycemia or catabolic features. Initiation of a basal insulin analog, such as glargine and detemir is the preferred regimen in these circumstances, typically at a dose of 0.1–0.2 units/kg/day, with titration based on fasting glucose and hypoglycemia occurrence. Ultra-long-acting analogs like glargine U300 and degludec provide even greater consistency and reduce glycemic variability, making them particularly suitable for older adults or people at increased risk of nocturnal hypoglycemia [1]. However, traditional basal insulin analogs require at least one daily injection, and, as such, can be cumbersome and frequently lead to poor adherence, resulting in challenges in diabetes management for many people with T2DM.

In response to these challenges, innovative insulin formulations have emerged, offering the possibility of once-weekly injections. Among the novel treatment options, insulin icodec and insulin efsitora alfa (efsitora, also known as insulin Fc, insulin BIF, or LY3209590), an Fc-fused insulin receptor agonist, mark a significant advancement in insulin therapy [2–6]. These insulin analogs aim to deliver consistent blood glucose control with only one subcutaneous injection per week. Insulin icodec has already been approved since 2024 and it is now available in Europe, Canada, Australia, and China for treatment of diabetes [7]. This is considered a significant departure from the daily injections required with conventional basal insulins, making diabetes management easier, more convenient and potentially more effective for people with T2DM, especially those who have struggled with a daily routine of multiple injections.

Individual randomized controlled trials (RCTs) have shown promising results regarding once-weekly insulin analogs, in terms of both efficacy and safety. By synthesizing data from all to-date available RCTs, this systematic review and meta-analysis aims to assess the efficacy and safety of once-weekly basal insulin analogs compared to daily basal insulin analogs.

## Methods

### Protocol and Registration

This systematic review adhered to the recommendations of the Preferred Reporting Items for Systematic Reviews and Meta-analyses (PRISMA) statement [8]. All research was conducted according to a protocol registered in the PROSPERO database (PROSPERO registration number: CRD420251060020, review protocol available at https://www.crd.york.ac.uk/PROSPERO/view/CRD420251060020).

### Information Sources and Searches

We systematically searched MEDLINE/PubMed, the Cochrane Library and Clinicaltrials.gov from database inception to June 5th, 2025. The search strategy included relevant free-text terms and Medical Subject Headings (MeSH), including “icodec”, “BIF”, “insulin Fc”, “LY3209590”, “insulin efsitora”, “efsitora alfa”, “weekly insulin”, “long acting insulin”, as detailed in Tables S1-S3. No language or date restrictions were applied. Additionally, relevant references from the included trials were reviewed for potential inclusion. In addition to bibliographic databases, ClinicalTrials.gov was searched to identify completed trials with publicly available results, consistent with current methodological recommendations [9]. Conference abstracts were not systematically searched or included in the present review.

### Study Selection

Study selection was conducted in two stages. First, titles and abstracts were screened to identify potentially eligible studies. Full texts of relevant articles were subsequently retrieved and assessed for eligibility according to predefined inclusion and exclusion criteria. Study selection was performed independently by two reviewers (D.R. and T.P.) using the Rayyan web-based systematic review software [10]. Disagreements between reviewers at any stage of the selection process were resolved through discussion; when necessary, a third reviewer was consulted to help resolve disagreements.

We included RCTs that met the following criteria: (a) investigated an once-weekly basal insulin analog (i.e., insulin icodec or insulin efsitora) as the intervention, (b) used a daily basal insulin analog as the comparator, (c) enrolled adults with T2DM, regardless of previous insulin treatment status, (d) had full results publicly available at the time of screening, (e) reported at least one outcome of interest. We excluded: (a) non-randomized studies, (b) phase 1 RCTs, (c) trials that included participants with any type of diabetes other than T2DM, (d) conference abstracts.

### Data Extraction

Data extraction was performed independently by two reviewers (D.R. and S.C.) using a pilot-tested Microsoft Excel data extraction form. Discrepancies between reviewers were resolved through discussion and consensus. When agreement could not be reached, a third reviewer was consulted to adjudicate and make the final decision.

The following data were extracted from each eligible study:

- Study and participant baseline characteristics.
- Intervention and comparator details: type of once-weekly basal insulin, type of daily basal insulin, treatment duration, and whether a loading dose was used.
- Outcomes:
  - The primary outcome was the mean change from baseline in HbA1c (%). Secondary continuous outcomes included change from baseline in time in range (TIR) (%) (70–180 mg/dl), fasting plasma glucose (FPG) (mg/dL), and body weight (kg). For these, we extracted the mean difference and standard error (SE) between the two treatment groups.
  - For dichotomous outcomes (achievement of HbA1c < 7.0%, and occurrence of level 2 or 3 hypoglycemia), we extracted the number of participants in whom the outcome occurred (e.g., the number of participants experiencing at least one event of level 2 or level 3 hypoglycemia) and the total number of participants analyzed.

Level 2 hypoglycemia was defined as clinically significant hypoglycemia, confirmed by glucometer measurement of blood glucose < 3.0 mmol/L (< 54 mg/dL), while level 3 hypoglycemia was defined as severe hypoglycemia characterized by cognitive impairment requiring external assistance for recovery [11].

### Risk of Bias Assessment in Individual Studies

Risk of bias was assessed using the Cochrane Risk of Bias 2 (RoB 2) tool for randomized controlled trials [12]. Two reviewers (D.R. and S.C.) independently evaluated the risk of bias for the primary outcome (change in HbA1c) for each included study. Each domain was judged as having “low risk”, “some concerns”, or “high risk”, with an overall risk of bias assigned accordingly. The overall risk of bias within a study was determined as low risk when all domains were rated low risk, as some concerns when at least one domain raised some concerns and no domains were rated high risk, and as high risk when at least one domain was judged high risk. Judgments were made based on the published reports and any supplementary materials or protocols. Discrepancies were resolved through discussion between the two reviewers. Remaining conflicts were resolved by consultation with a third reviewer.

### Statistical Analysis

We did meta-analyses of mean differences for continuous outcomes and odds ratios for dichotomous outcomes, each reported with the respective 95% confidence interval (CI). Statistical significance was determined using two-tailed p-values, with a threshold of 0.05. Between-study heterogeneity was assessed using the I² statistic. I² values ≤ 40% were considered as unimportant heterogeneity, 30% ≤ I² ≤60% as moderate heterogeneity, 50% ≤ I² ≤90% as substantial heterogeneity, and 75% ≤ I² ≤100% as considerable heterogeneity [13]. Given the expected variability among studies, a random-effects model (DerSimonian and Laird method) was applied in all analyses [14]. Pre-specified subgroup analyses were performed for the primary outcome, based on insulin-naivety status at baseline, type of intervention (once-weekly basal insulin analog), as well as type of comparator (daily basal insulin analog). Subgroup differences were examined using the test for subgroup interaction and statistical significance was set at *p* < 0.10 for subgroup interaction. We also performed a post hoc sensitivity analysis for HbA1c and FPG excluding two trials with titration algorithms that differed from the other included trials: one in which the efsitora arms were titrated less frequently and to higher fasting glucose targets than the degludec arm [15], and another in which efsitora was administered using a mg-based individualized dosing algorithm based on fasting glucose and body weight [16]. All analyses were conducted using the meta package in R (version 2025.9.2.418).

When multiple follow-up time points were reported within a trial for the same outcome, data from the longest available follow-up duration were preferentially extracted and included in the meta-analysis. For each outcome at the selected time point, efficacy data were extracted according to the estimand definitions prespecified by the original study investigators. Across trials, estimands varied and included efficacy or trial-product estimands (estimating hypothetical treatment effects assuming continued adherence to the randomized intervention without rescue therapy) as well as treatment policy or treatment-regimen estimands (estimating between-group effects among all randomized participants regardless of treatment adherence, rescue medication use, or changes in background therapy). When more than one estimand was reported for the same outcome and follow-up duration, results corresponding to the efficacy estimand specified in the main trial publication were preferentially extracted. In some studies, TIR was reported as the mean percentage of time in range during a predefined assessment period, whereas in other trials it was reported as change from baseline. Furthermore, for multi-arm studies evaluating continuous outcomes, we adjusted the standard errors of pairwise comparisons to account for shared control groups and avoid double counting [17]. For dichotomous outcomes, multiple intervention arms were combined into a single intervention group by summing the number of participants with the outcome of interest and the number of participants analyzed across intervention arms.

### Risk of Bias due to Missing Results

We assessed small-study effects among included studies for the primary outcome using visual inspection of funnel plots and Egger’s regression test [18], when data for at least ten studies were available, to explore potential publication bias [19]. Asymmetry in the funnel plot or a significant Egger’s test (i.e., *p* < 0.10) were considered as an indication of possible publication bias [20].

## Results

### Search Results and Study Selection

The searches in MEDLINE/PubMed (*n* = 1,721), Cochrane Library (*n* = 2,253) and Clinical Trials database (*n* = 125) identified 4,099 records. After removal of 462 duplicates, 3,637 records underwent title and abstract screening, with 3,597 records excluded based on predefined eligibility criteria. A total of 40 full-text articles were assessed for eligibility. Of these, 26 were excluded due to reasons including ineligible study type (*n* = 21), ineligible study population (people with type 1 diabetes) (*n* = 2), or no publicly available results (*n* = 3). Finally, 14 RCTs involving 8,487 participants were deemed eligible for inclusion [2, 5, 15, 16, 21–30] (Fig. S1).

### Study Characteristics

The main characteristics of the included studies are presented in Table 1. One study was published in 2020, two studies in 2021, seven studies in 2023, one study in 2024, and three studies in 2025. Eight studies assessed once-weekly insulin icodec [2, 21–27], and six studies assessed once-weekly insulin efsitora [5, 15, 16, 28–30]. All studies compared a once-weekly basal insulin with a daily basal insulin, such as glargine U100, glargine U300 or degludec, with or without prandial insulin. One trial evaluated two, and one trial evaluated three titration regimens for icodec [22, 23], while one study evaluated two different titration algorithms for efsitora [15]. Data from these arms were not combined and were rather used as different entries in the analyses. Seven trials included insulin-naive participants [2, 5, 16, 21, 23, 25, 27]. Study durations varied, with six trials lasting 26 weeks, four lasting 52 weeks, two lasting 16 weeks, one lasting 32 weeks, and one extending to 78 weeks. Eleven studies were open-label, while three were double-blind. Background glucose-lowering therapies prior to randomization varied across studies and included basal insulin, with or without prandial insulin, with or without other oral or injectable antidiabetics (metformin, DPP-4 inhibitors, SGLT-2 inhibitors, sulfonylureas, GLP-1 RAs, thiazolidinediones) [15, 22, 24, 26, 28, 29], or various combinations of non-insulin medications [2, 5, 16, 21, 23, 25, 27, 30]. In trials where participants were already receiving basal insulin therapy at baseline, pre-randomization basal insulin was discontinued at randomization and replaced by the study-assigned basal insulin regimen (once-weekly or daily), according to the trial protocol. Participants’ mean HbA1c at baseline was 8.28% across all trials, mean age was 59.3 years and mean duration of diabetes was 12.9 years. In total, 2,265 individuals were randomized to receive insulin icodec, 2,293 randomized to receive insulin efsitora, and 3,929 to receive daily basal insulin (glargine or degludec).

**Table 1:** Βaseline characteristics of included studies

Author, year; trial identification	Study duration, weeks	Blinding status	Background glucose-lowering therapy prior to randomization	Study arms	No. of participants randomized	HbA1c, %	Body weight, kg	Diabetes duration, years	Age, years
Rosenstock, 2020; NCT03751657 [18]	26	Double-blind	Metformin monotherapy with (46.6%) or without DDP-4i	Icodec 70U	125	8.09±0.70	89.7±16.5	10.5±8.4	59.7±8.2
				Glargine U100 10U	122	7.96±0.65	91.3±15.7	8.8±6.1	59.4±9.5
Bajaj, 2021; NCT03922750 [19]	16	Open-label	Once or twice daily basal insulin, along with metformin (41.6%), with (22.1%) or without DPP-4i, with (19.5%) or without SGLT-2i, or with both DDP-4i and SGLT-2i (16.2%)	Icodec LD (variable dose)	54	7.8±0.7	NA	13.8±7.7	62.4±7.2
				Icodec NLD (variable dose)	50	7.9±0.7	NA	16.8±8.2	62.1±8.2
				Glargine U100 (variable dose)	50	7.9±0.7	NA	14.8±8.1	60.5±7.9
Lingvay, 2021; NCT03951805 [20]	16	Double-blind	Metformin monotherapy (38%), metformin plus SGLT-2i (21.5%), metformin plus DPP-4i (28.3%) or metformin plus SGLT-2i and DPP-4i (12.2%)	Icodec 70U (Titration Algorithm A)	51	8.0±0.7	91.4±17.6	9.8±7.2	59.8±9.1
				Icodec 70U (Titration Algorithm B)	51	8.1±0.8	90.4±18.0	9.6±4.9	61.2±8.0
				Icodec 70U (Titration Algorithm C)	52	8.2±0.9	87.3±14.0	9.2±4.4	61.4±8.0
				Glargine U100 10U	51	8.2±0.8	86.4±17.1	11.8±6.8	60.2±8.1
Rosenstock, 2023; ONWARDS 1, NCT04460885 [2]	78	Open-label	Metformin (90.0%), GLP-1 RA (17.8%), SGLT-2i (36.5%), sulfonylurea (45.3%), DPP-4i (35.4%), thiazolidinediones (5.0%), α-Glucosidase inhibitor (4.6%), glinides (2.6%)	Icodec 70U	492	8.5±1.0	85.2±17.7	11.6±6.7	59.1±10.1
				Glargine 10U	492	8.4±1.0	84.3±17.6	11.5±6.8	58.9±9.9
Philis-Tsimikas, 2023, ONWARDS 2, NCT04770532 [21]	26	Open-label	Once-daily or twice-daily basal insulin, with or without non-insulin glucose-lowering agents	Icodec (variable dose)	263	8.17±0.77	83.7±18.4	16.5±8.4	62.3±9.8
				Degludec	263	8.10±0.77	81.5±17.1	16.5±8.4	62.6± 8.4
Lingvay, 2023; ONWARDS 3, NCT04795531 [22]	26	Double-blind	Any noninsulin glucose-lowering medication	Icodec 70U and once-daily placebo	294	8.55±1.11	85.8±20.1	10.33±6.63	58±10
				Degludec 10U and once-weekly placebo	294	8.48±1.01	83.2±18.2	11.03±7.30	59±10
Mathieu, 2023; ONWARDS 4, NCT04880850 [23]	26	Open-label	Basal-bolus insulin regimen with or without non-insulin antidiabetics	Icodec (variable dose) with aspart	291	8.29±0.86	85.5±17.6	18.0±9.1	59.7±10.1
				Glargine U100 with aspart	291	8.31±0.90	83.1±17.3	16.3±7.7	59.9±9.9
Frias, 2023; NCT03736785 [24]	32	Open-label	Basal insulin (glargine or detemir or degludec) with or without up to three oral antidiabetic medications	Efsitora group 1	135	8.2±0.9	90.6±19.5	15.0±8.5	60.2±9.9
				Efsitora group 2	132	8.0±0.9	88.1±18.9	14.1±9.1	59.6±11.3
				Degludec	132	8.1±0.9	87.1±20.7	15.1±8.0	60.8±10.0
Bajaj, 2023; ONWARDS 5, NCT04760626 [25]	52	Open-label	Any noninsulin glucose-lowering medication	Icodec 70U	542	8.96±1.6	93.2±22.5	11.9±6.9	59.1±10.8
				Degludec,glargine U100,glargine U300	543	8.88±1.5	94.3±21.5	12.0±7.6	59.4±10.2
Bue-Valleskey, 2023; NCT04450394 [26]	26	Open-label	Metformin monotherapy (57.9%), metformin plus SGLT-2i (20.1%), metformin plus DPP-4i (12.6%) or metformin plus SGLT-2i and DPP-4i (9.4%)	Efsitora 3.0-16.5 mg	143	8.1 ± 0.8	91.0 ± 20.8	10.4 ± 6.8	57.3 ± 9.7
				Degludec 10U	135	8.0 ± 0.8	90.6 ± 19.6	9.7 ± 6.0	59.4 ± 9.1
Wysham, 2024; QWINT-2, NCT05362058 [5]	52	Open-label	Metformin (83.9%), GLP-1 RA (50.0%), SGLT-2i (35.3%), sulfonylurea (32.8%), DPP-4i (16.4%), thiazolidinediones (5.2%), α-Glucosidase inhibitor (4.0%)	Efsitora one-time dose of 300U, followed by a starting dose of 100U	466	8.21±0.96	86.83±20.53	11.78±7.54	57.6±10.6
				Degludec 10U	462	8.23±0.96	86.12±18.93	11.42±6.97	57.3±11.0
Rosenstock, 2025; QWINT-1,NCT05662332 [29]	52	Open-label	Metformin (93.5%), DPP-4i (31.1%), SGLT-2i (22.1%), GLP-1 RA (9.7%), thiazolidinedione (5.2%)	Efsitora 100U for 4 weeks, followed by 150, 250 or 400U weekly	397	8.20±0.91	89.3±19.2	9.2±6.6	56.4±10.0
				Glargine 10U, escalated as needed	398	8.27±1.07	85.5±19.7	9.6±6.9	56.2±9.7
Philis-Tsimikas, 2025; QWINT-3, NCT05275400 [27]	78	Open-label	Basal insulin and up to three non-insulin medications	Efsitora	655	7.7±0.96	83.6±18.59	14.5±8.52	62.0±10.37
				Insulin degludec	331	7.7±0.89	84.3±18.74	14.4±7.04	62.0±9.63
Blevins, 2025; QWINT-4, NCT05462756 [28]	26	Open-label	Basal and prandial insulin and up to three non-insulin medications	Efsitora and prandial insulin lispro (variable doses)	365	8.36±0.7	87.8±19.9	16.6±8.8	58.3±10.5
				Insulin glargine U100 and prandial insulin lispro (variable doses)	365	88.36±0.80	88.4±19.7	16.9±9.0	59.4±10.5

Data for HbA1c, body weight, diabetes duration and age are presented as mean ± SD values

GLP-1 RA, glucagon-like peptide-1 receptor agonist; DDP-4i, dipeptidyl peptidase-4 inhibitor; LD, loading dose; NA, not available; NLD, no loading dose; SGLT-2i, sodium-glucose cotransporter-2 inhibitor; U, units

The results of the risk of bias assessment for each included study are summarized in Table S4. Overall, 12 RCTs were rated as having low overall risk of bias. One study was judged to have high risk of bias [16], while one trial was judged to have some concerns [31].

### HbA1c

**Fig. 1:**
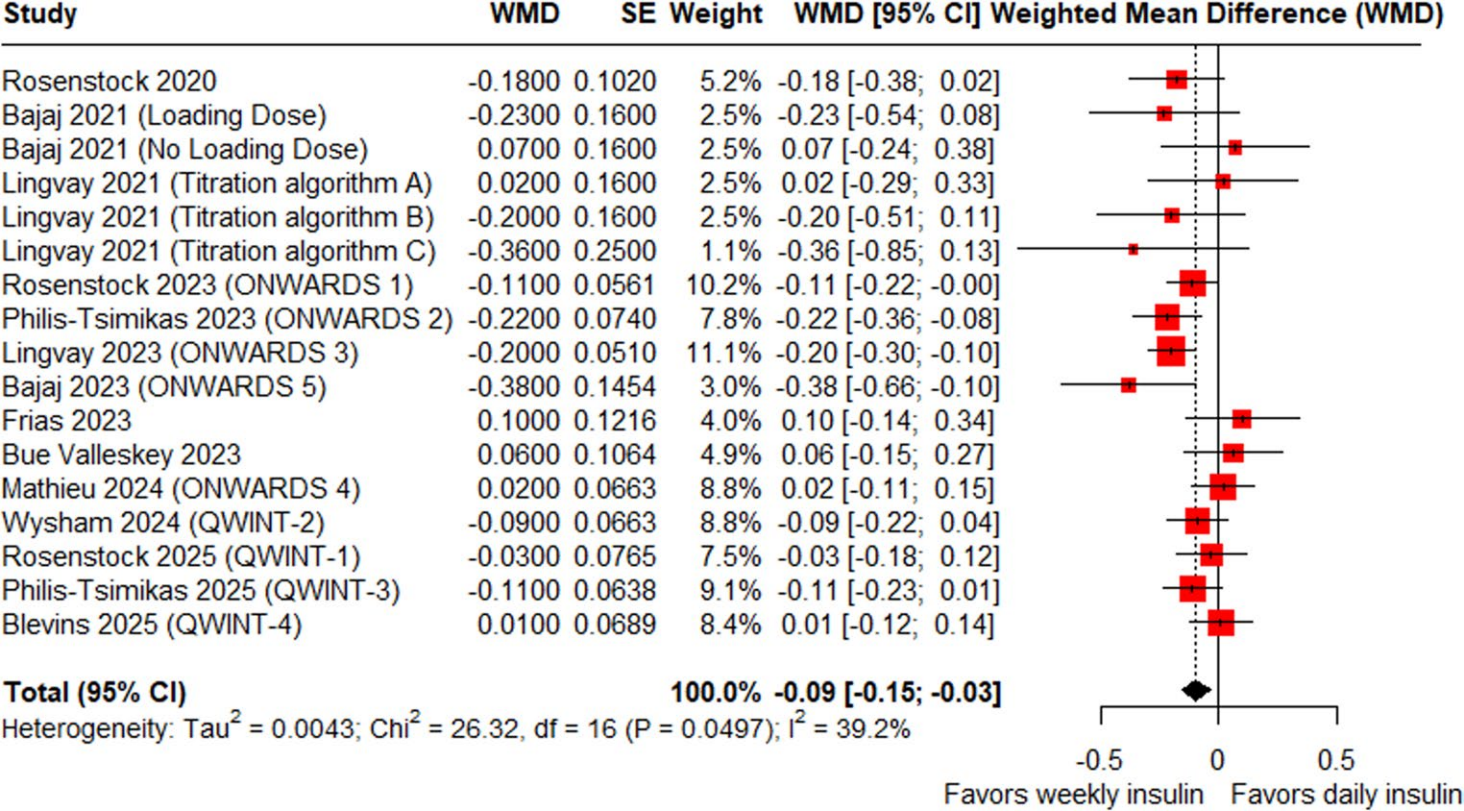
Difference in mean change in HbA1c between once-weekly and daily basal insulin analogs

A total of 14 RCTs (8,487 participants) were included in the meta-analysis for the mean change in HbA1c from baseline. Once-weekly basal insulin analogs reduced HbA1c (-0.09% [95% CI -0.15 to -0.03], I^2^ 39.2%) compared to daily basal insulin analogs (Fig. 1). Furthermore, once-weekly basal insulin showed higher odds of achieving an HbA1c < 7.0% (OR 1.32 [95% CI 1.07 to 1.63], I^2^ 64.9%) compared to daily basal insulin (Fig. S2).

The subgroup analysis based on insulin naivety status revealed no subgroup effect in the change in HbA1c (p for interaction = 0.24) (Fig. S3). The subgroup analysis based on type of intervention, indicated a subgroup effect favoring insulin icodec over insulin efsitora (p for interaction = 0.03) (Fig. S4). There was no subgroup effect based on type of comparator (insulin glargine or degludec, p for interaction = 0.40) (Fig. S5). Of note, in this subgroup analysis, ONWARDS 5 was not included, since the comparator arm included both insulin glargine and insulin degludec. A post hoc sensitivity analysis excluding two trials with trial-specific dosing/titration algorithms [15, 16] resulted in an effect estimate of -0.11% (95% CI -0.17 to -0.05; I² 33.7%) (Fig. S6).

### Time in Range

A total of 10 RCTs (5,741 participants) were included in the meta-analysis of TIR. Of the included studies, four reported the mean change in TIR from baseline [5, 21–23], whereas the remaining trials reported mean TIR over the specified assessment period without a baseline reference value. TIR was defined as the proportion of time spent with glucose concentrations within the predefined target range 70 and 180 mg/dL. One study reported slightly modified (71–180 mg/dL) [15] target range and another one a narrower (70–140 mg/dL) [21] target range; the latter was not included in the main analysis. In the pooled analysis, participants treated with once-weekly basal insulin achieved higher TIR (1.86% [95% CI, 0.73 to 2.98], I^2^ 34%) (Fig. S7). We also performed a post hoc sensitivity analysis, including the study which defined TIR as 70–140 mg/dL and TIR was higher again in once-weekly basal insulin compared to daily basal insulin (2.05% [95% CI, 0.91 to 3.19]) (Fig. S8), confirming the robustness of the primary findings.

### Fasting Plasma Glucose

Thirteen RCTs (7,402 participants) were included in the meta-analysis evaluating the mean change in FPG from baseline. No difference was observed between the treatment arms (0.01 mg/dL [95% CI -2.65 to 2.67], I^2^ 74.7%) (Fig. S9).

A post hoc sensitivity analysis excluding two trials with trial-specific dosing/titration algorithms [15, 16] also showed no difference between once-weekly and daily basal insulin analogs (MD -0.22 mg/dL, 95% CI -0.47 to 0.04; I² 16.3%) (Fig. S10).

### Body Weight

Fourteen RCTs (8,487 participants) were included in the meta-analysis evaluating the mean change in body weight from baseline. Meta-analysis showed that once-weekly basal insulin analogs were associated with a small increase in body weight compared to daily basal insulin analogs (0.34 kg [95% CI 0.07 to 0.61], I^2^ 38%) (Fig. S11).

### Level 2 or 3 Hypoglycemia

Fourteen RCTs (8,088 participants) were included in the meta-analysis for level 2 or 3 hypoglycemia. The odds of experiencing level 2 or 3 hypoglycemia were similar between once-weekly and daily basal insulin analogs (OR 1.14 [95% CI 0.92 to 1.40], I^2^ 49.1%), indicating no difference in clinically important hypoglycemia risk (Fig. S12).

### Risk of Bias Due To Missing Results (Publication Bias)

Publication bias was evaluated for the primary outcome of change in HbA1c using a funnel plot and Egger’s regression test (Fig. S13). Visual inspection of the funnel plot revealed a symmetrical distribution of effect sizes, thus indicating a low risk for publication bias. Egger’s test showed no significant asymmetry (z= -0.38, *p* = 0.70), further supporting no evidence of small-study effects.

## Discussion

This systematic review and meta-analysis provides an up-to-date and comprehensive synthesis of RCTs comparing once-weekly basal insulin analogs icodec and efsitora with daily basal insulin analogs glargine or degludec in adults with T2DM. A total of 14 RCTs including 8,487 participants were analyzed. Overall, once-weekly basal insulin analogs were associated with a small reduction in HbA1c compared with daily basal insulin analogs, a modest improvement in TIR, a small increase in body weight, and no difference in the risk of clinically significant hypoglycemia.

The magnitude of HbA1c reduction observed in favor of weekly insulin (-0.09%) is unlikely to be clinically meaningful on its own. Nonetheless, even small improvements in glycemic parameters may be relevant at the population level, particularly when achieved without an increase in hypoglycemia [11, 32, 33]. Importantly, the improvement in TIR, although modest, supports the consistency of glycemic control achieved with weekly formulations. Taken together, these findings suggest that once-weekly basal insulin analogs provide glycemic efficacy that is at least comparable to that of daily basal insulin analogs.

This systematic review adds to the existing literature in several ways [34–39]. First, it includes the most recent phase 3 trials, including the full QWINT program and the latest ONWARDS studies, thereby providing an updated synthesis. Second, it incorporates both once-weekly basal insulin analogs (icodec and efsitora) within the same analytical framework. Third, we appropriately accounted for multi-arm trial designs, avoiding double counting of shared control groups, and data were preferentially extracted at the longest available follow-up to reflect sustained treatment effects. Furthermore, estimand definitions were carefully considered, and efficacy estimands were preferentially used when multiple estimands were reported. Our findings are broadly consistent with prior systematic reviews and meta-analyses, which have generally reported noninferiority or modest superiority of once-weekly basal insulin analogs in terms of HbA1c reduction and similar rates of hypoglycemia [34–39]. The observed small reduction in HbA1c and higher odds of achieving HbA1c < 7% in the present analysis align with previous reports. Data on weight change across studies have been inconsistent in earlier syntheses [34–39]; in our analysis, once-weekly insulin was associated with a small increase in body weight compared with daily basal insulin, although the absolute magnitude was small and unlikely to be clinically relevant.

Subgroup analyses did not demonstrate a significant interaction according to baseline insulin-naïve status, suggesting that weekly basal insulin analogs provide similar glycemic benefit in both insulin-naïve and previously insulin-treated individuals. This finding indicates that prior insulin exposure does not appear to modify the comparative efficacy of once-weekly versus daily basal insulin analogs. A subgroup effect was observed according to the type of once-weekly insulin, with icodec showing a slightly greater HbA1c reduction compared with efsitora. However, this finding should be interpreted cautiously, as subgroup analyses are observational within trials and may be influenced by differences in study design, populations, and comparator regimens. No subgroup differences were identified according to the type of daily basal insulin analog in the comparator arm (glargine or degludec).

With respect to safety, no difference was observed in the risk of level 2 or 3 hypoglycemia between once-weekly and daily basal insulin analogs. This finding is clinically important, as concerns regarding prolonged pharmacodynamic exposure with ultra-long-acting insulin formulations could theoretically raise safety considerations [6]. The absence of increased clinically significant hypoglycemia provides reassurance regarding the safety profile of once-weekly formulations within the durations studied.

From a clinical perspective, the potential advantage of once-weekly basal insulin analogs lies not in a large incremental glycemic effect, but in simplification of treatment. Reduction in injection frequency from 365 to 52 injections per year may meaningfully reduce treatment burden for many individuals with T2DM. Treatment complexity and injection frequency are well-recognized contributors to non-adherence, and missed or mistimed insulin doses are associated with poorer glycemic control [40]. Although adherence and patient-reported outcomes were not assessed in the present review, simplification of the regimen may translate into improved persistence and satisfaction in real-world settings. Such potential benefits warrant further dedicated investigation.

While the reduced injection frequency with once-weekly basal insulin may improve adherence, the transition from daily to weekly regimens may introduce some considerations. Calculating equivalent weekly doses, including the use of loading doses, may increase regimen complexity, particularly for patients switching from daily basal insulin therapy. Careful dose titration and patient education are therefore important to ensure safe and effective implementation. These factors should be weighed alongside the potential benefits of reduced injection burden when considering once-weekly basal insulin analogs in clinical practice.

Beyond treatment burden, the role of once-weekly basal insulin must also be considered within the evolving therapeutic landscape of T2DM. In the contemporary era of glucagon-like peptide-1 receptor agonist (GLP-1 RA) use, daily basal insulin has increasingly shifted from first-line injectable therapy to a complementary option when GLP-1 RAs are insufficient, contraindicated, or when patients present with markedly elevated HbA1c levels [41]. The complementary mechanisms of once-weekly basal insulin analogs and GLP-1 RAs [42] provide a strong rationale for combination strategies, although such approaches were beyond the scope of the present analysis. The development of once-weekly fixed-ratio combinations, such as insulin icodec with semaglutide (IcoSema), further emphasizes the potential for simplified regimens that simultaneously improve glycemic control, reduce hypoglycemia risk, and mitigate insulin-associated weight gain [43–46]. Future studies should clarify the role of these combination strategies in optimizing metabolic outcomes while preserving the treatment-burden advantages of once-weekly therapy.

Specific limitations should be considered when interpreting our findings. First, although ClinicalTrials.gov was searched, conference abstracts were not systematically searched or included. While searching conference abstracts is considered highly desirable, albeit not mandatory, in comprehensive evidence syntheses [9], they frequently lack sufficient detail regarding outcomes and methodological characteristics to permit reliable quantitative synthesis [47–49]. The decision to exclude them was based on methodological research indicating that conference abstracts are frequently insufficiently reported with respect to outcome data and study details, which may preclude reliable data extraction and quantitative synthesis, and that their inclusion may have limited impact on pooled effect estimates in fields with an already substantial body of full-text evidence [50]. Nevertheless, exclusion of conference abstracts may limit completeness and may contribute to potential publication bias. Although funnel plot inspection and Egger’s test did not suggest small-study effects, such analyses evaluate asymmetry among included studies and cannot fully account for unpublished or selectively reported data. Second, heterogeneity across studies was moderate for several outcomes. Differences in baseline characteristics (including diabetes duration and baseline HbA1c), background glucose-lowering therapies, use of loading doses, open-label versus double-blind design, and study duration may have contributed to variability in effect estimates. Third, reporting of TIR differed across trials, with some studies reporting change from baseline and others reporting mean TIR during a predefined assessment period. Finally, patient-reported outcomes, treatment satisfaction, and quality-of-life measures were outside the scope of this review. Given that regimen simplification is a central rationale for once-weekly basal insulin, the paucity of systematic synthesis of patient-centered outcomes represents an important gap.

Future research should focus on longer-term durability of glycemic control with once-weekly basal insulin analogs, direct head-to-head comparisons between different weekly formulations, and real-world studies evaluating adherence, persistence, health-related quality of life, and country-specific cost-effectiveness. As clinical experience with once-weekly basal insulin expands, further evaluation in diverse populations, including older adults and individuals with comorbidities, will also be important.

## Conclusion

Our systematic review and meta-analysis including both icodec and efsitora demonstrates that once-weekly basal insulin analogs improve HbA1c and TIR without increasing clinically significant hypoglycemia compared to daily basal insulin analogs, although a small increase in body weight was observed. While the absolute glycemic benefit is modest, simplification of injection frequency may offer meaningful practical advantages. Once-weekly basal insulin analogs therefore represent a promising alternative to daily basal insulin analogs, with potential to reduce treatment burden while maintaining efficacy and safety.

## Key References

- Rosenstock J, Bain SC, Gowda A, Jódar E, Liang B, Lingvay I, et al. Weekly Icodec versus Daily Glargine U100 in Type 2 Diabetes without Previous Insulin. N Engl J Med. 2023;389:297–308. 10.1056/NEJMoa2303208.
  - ⚬ First phase 3 trial showing superiority rather than just non-inferiority.
- Karakasis P, Patoulias D, Pamporis K, Popovic DS, Stachteas P, Bougioukas KI, et al. Efficacy and safety of once-weekly versus once-daily basal insulin analogues in the treatment of type 2 diabetes mellitus: A systematic review and meta-analysis. Diabetes Obes Metab. 2023; 10.1111/dom.15259.
  - ⚬ First meta-analysis including both icodec and efsitora, including 3,962 patients from 9 RCTs.
- Wysham C, Bajaj HS, Del Prato S, Franco DR, Kiyosue A, Dahl D, et al. Insulin Efsitora versus Degludec in Type 2 Diabetes without Previous Insulin Treatment. N Engl J Med. 2024; 10.1056/NEJMoa2403953.
  - ⚬ This represents the first phase 3 evidence for efsitora in insulin-naive patients.
- Philis-Tsimikas A, Bergenstal RM, Bailey TS, Jinnouchi H, Thrasher JR, Ilag L, et al. Once-weekly insulin efsitora alfa versus once-daily insulin degludec in adults with type 2 diabetes currently treated with basal insulin (QWINT-3): a phase 3, randomised, non-inferiority trial. Lancet Lond Engl. 2025;405:2279–89. 10.1016/S0140-6736(25)01044-X/
  - ⚬ The longest trial in the QWINT program (78 weeks).

## Electronic Supplementary Material

Below is the link to the electronic supplementary material.

Supplementary Material 1 (DOCX 320 KB)

## Data Availability

No datasets were generated or analysed during the current study.

## Abbreviations

FPG: Fasting plasma glucose
HbA1c: Glycated hemoglobin
RCT: Randomized controlled trial
T1DM: Type 1 diabetes mellitus
T2DM: Type 2 diabetes mellitus
TIR: Time in range
