# Supplementary material for: Efficacy and safety of once-weekly basal insulin analogs versus daily basal insulin analogs in adults with type 2 diabetes: a systematic review and meta-analysis
Source: Curr Diab Rep. 2026 Jun 12;26(1):16. doi: 10.1007/s11892-026-01628-3 (PMC13260229; doi:10.1007/s11892-026-01628-3)
Supplement: Supplementary file 1 — Supplementary Material 1 (DOCX 320 KB) [file 11892_2026_1628_MOESM1_ESM.docx]

## **Fig. S1** Preferred Reporting Items for Systematic Reviews and Meta-Analyses (PRISMA) flow diagram.

**Identification of studies via databases and registers**

Records identified from:

MEDLINE (n=1,721)

Cochrane Library (n = 2,253)

Clinical Trials (n=125)

Registers (n = 0)

Records removed *before screening*:

Duplicate records removed

(n = 462)

Records marked as ineligible by automation tools (n = 0)

Records removed for other reasons (n = 0)

**Identification**

Records excluded

(n = 3,597)

Records screened

(n = 3,637)

Reports not retrieved

(n =0)

Reports sought for retrieval

(n = 40)

**Screening**

Reports excluded:

Study type (n = 21)

Type 1 diabetes (n = 2)

No publicly available results (n = 3)

Reports assessed for eligibility

(n =40)

Studies included in review

(n = 14)

**Included**

| **Fig. S2** Odds ratio of achieving an HbA1c <7.0% between once-weekly and daily basal insulin analogs. |
| --- |
| 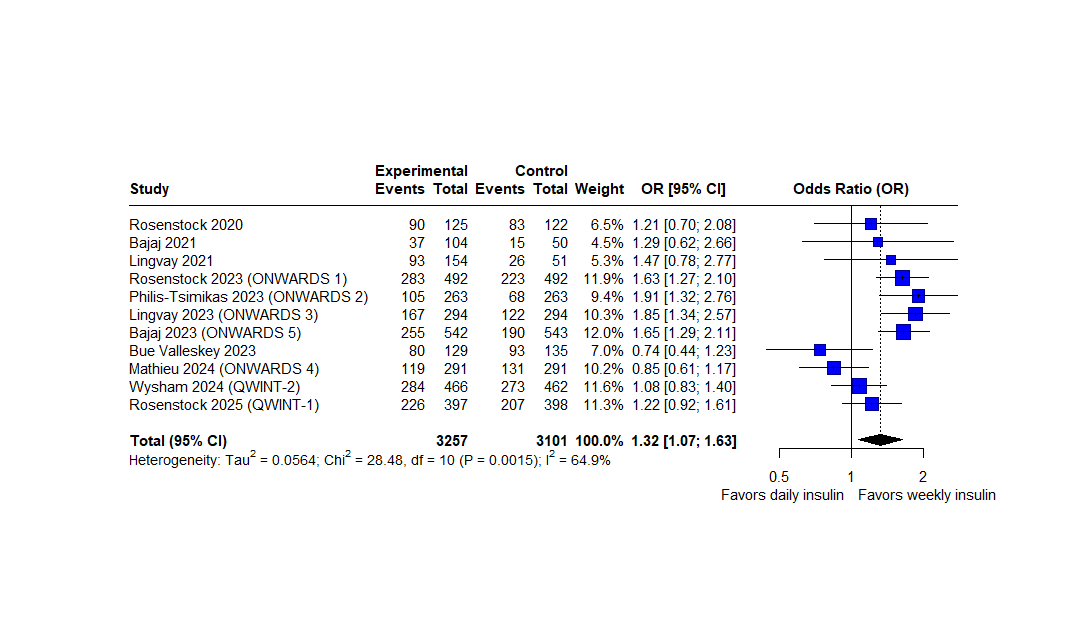 |

| **Fig. S3** Difference in mean change in HbA1c (%) between once-weekly and daily basal insulin analogs, based on insulin naivety status. |
| --- |
| 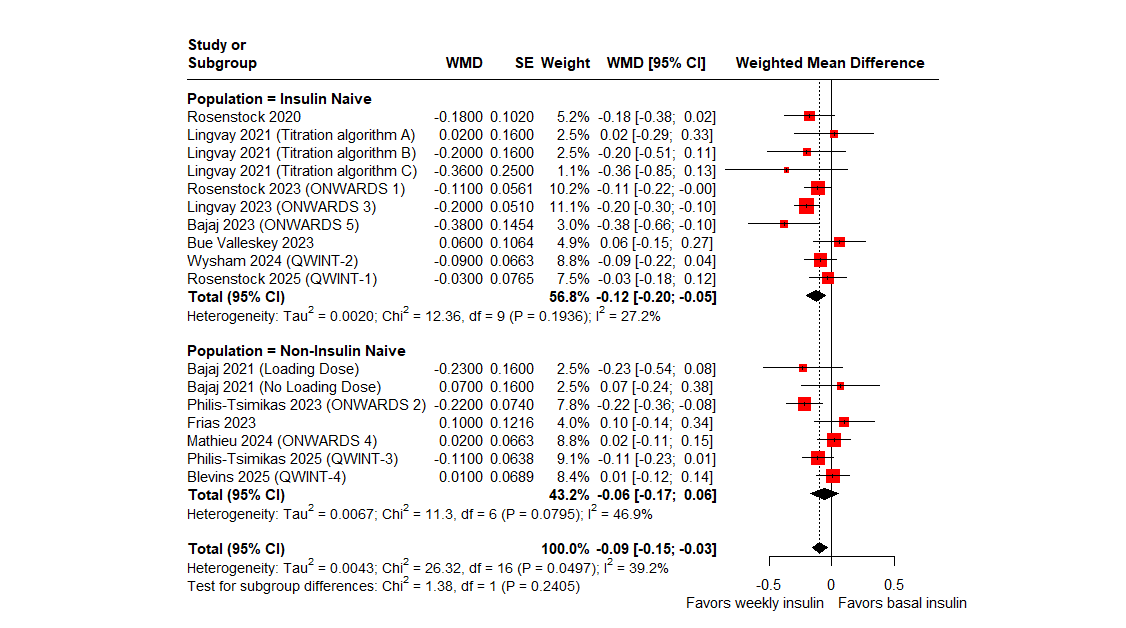 |

| **Fig. S4** Difference in mean change in HbA1c (%) between once-weekly and daily basal insulin analogs, based on intervention (type of once-weekly basal insulin analog) used. |
| --- |
| 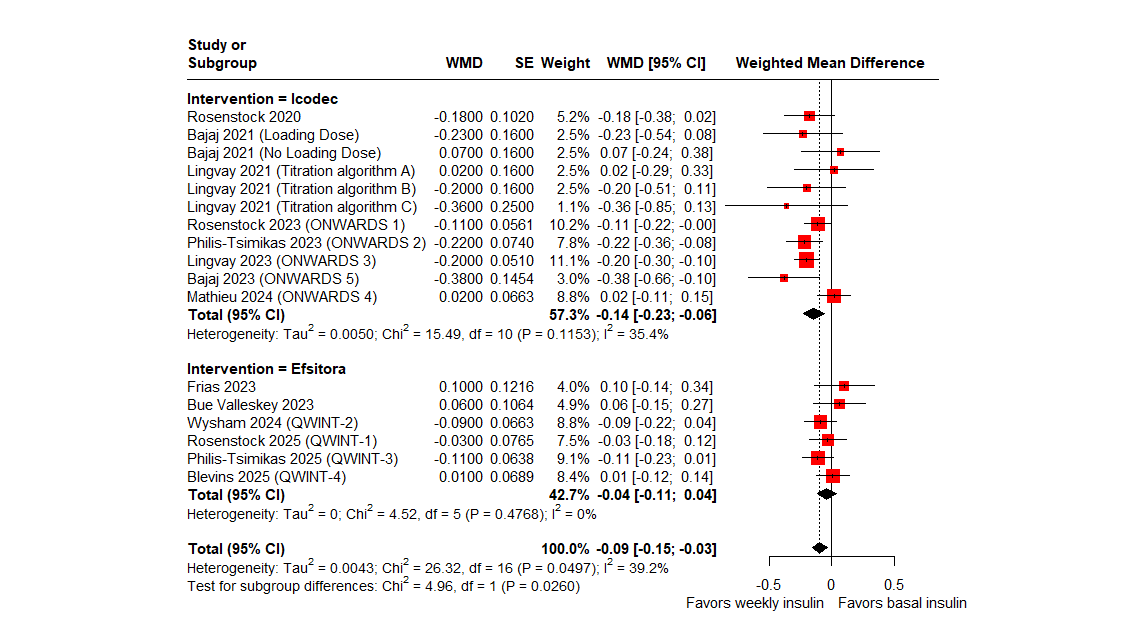 |

| **Fig. S5** Difference in mean change in HbA1c (%) between once-weekly and daily basal insulin analogs, based on comparator (type of daily basal insulin analog) used^a^. |
| --- |
| ^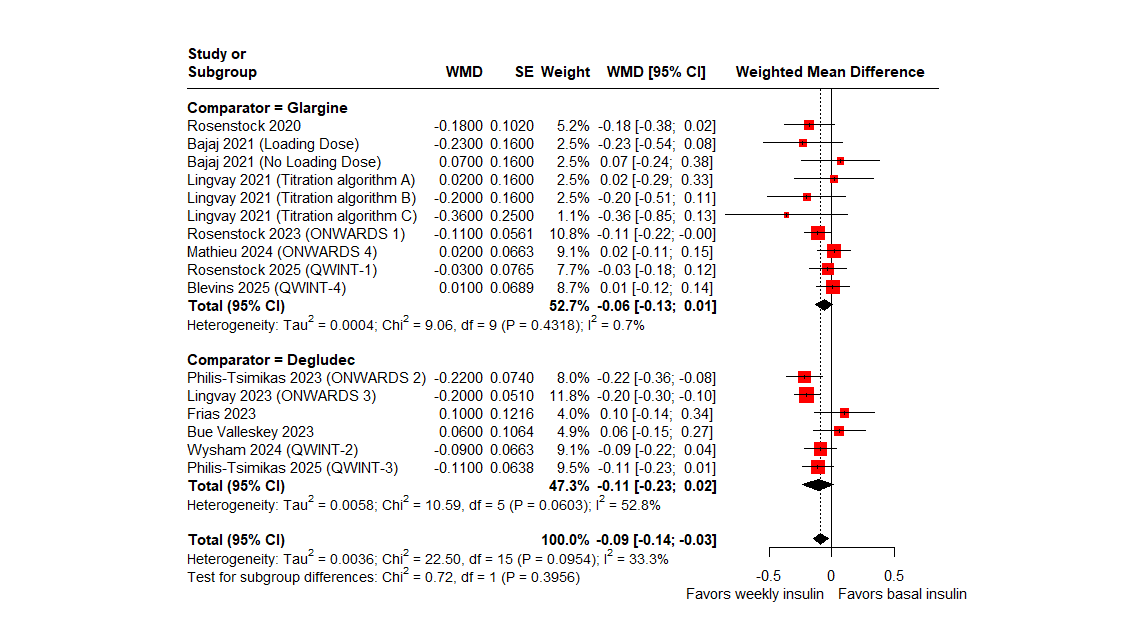^ |
| ^a^ONWARDS 5 was excluded from this analysis; comparator arm included both types of basal insulin and treatment allocation could not be determined. |

| **Fig. S6** Post hoc sensitivity analysis for the mean change in HbA1c (%) between once-weekly and daily basal insulin analogs, excluding two trials with trial-specific dosing/titration algorithms. |
| --- |
| 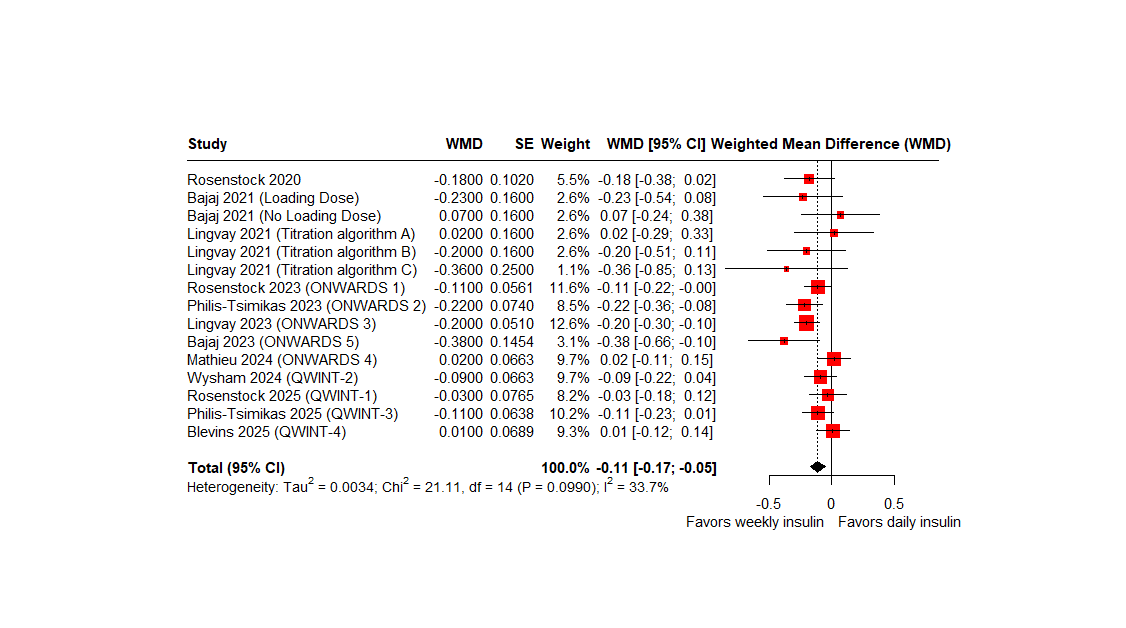 |
| ^a^Studies Bue Valleskey 2023 and Frias 2023 were excluded. |

| **Fig. S7** Difference in mean change in TIR between once-weekly and daily basal insulin analogs (main analysis). |
| --- |
| 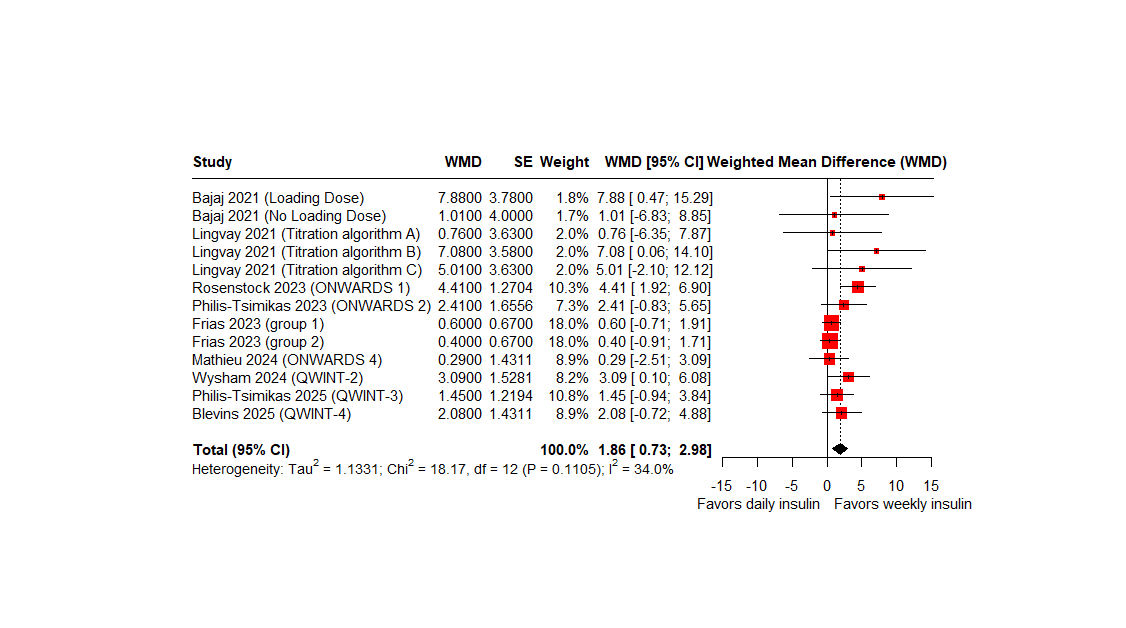 |

| **Fig. S8** Post hoc sensitivity analysis for the difference in mean change in TIR between once-weekly and daily basal insulin analogs, including a trial with different definition of TIR^a^. |
| --- |
| 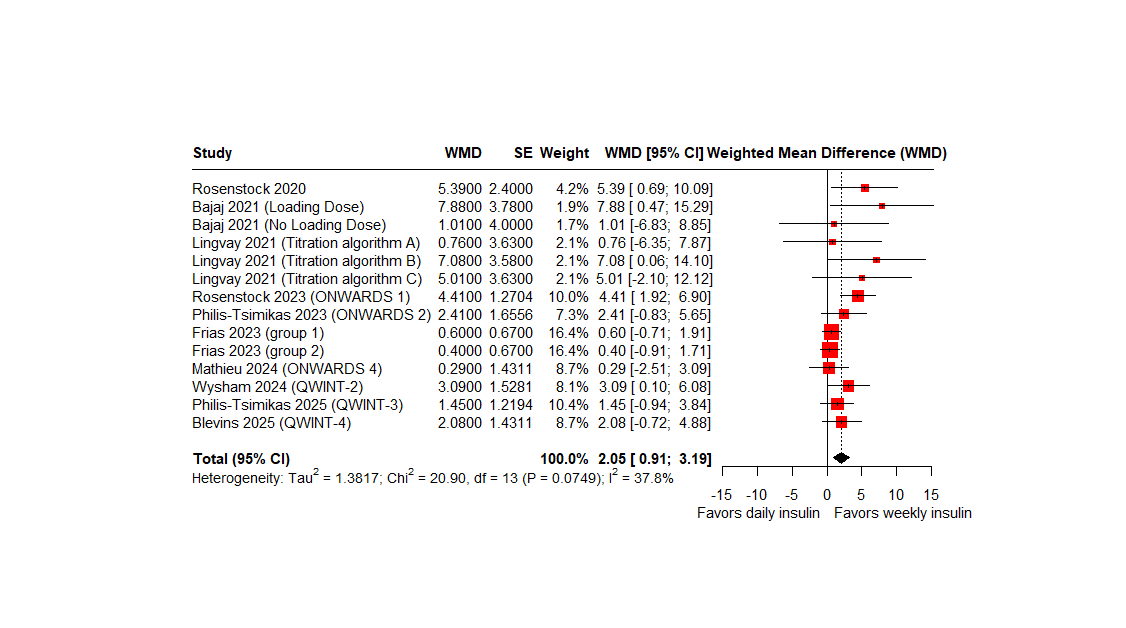 |
| ^a^TIR was defined generally as 70-180 mg/dL, except for Rosenstock 2020 (70-140 mg/dL) and Frias 2023 (71-180 mg/dL) |

| **Fig. S9** Difference in mean change in FPG (mg/dL) between once-weekly and daily basal insulin analogs (main analysis). |
| --- |
| 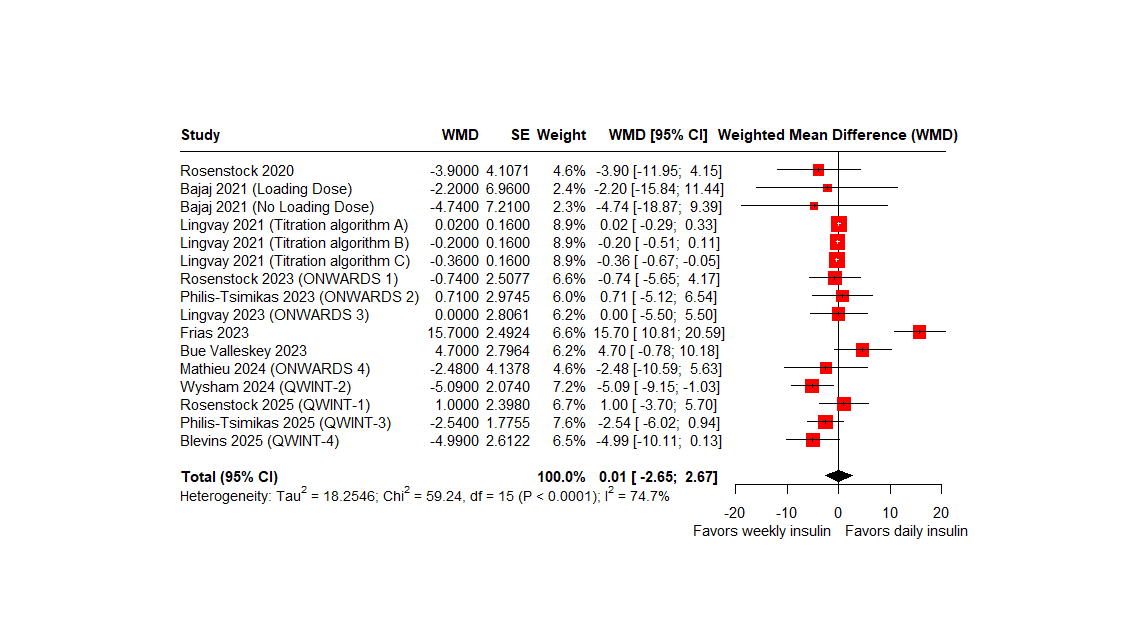 |

| **Fig. S10** Post hoc sensitivity analysis for the mean change in FPG (mg/dL) between once-weekly and daily basal insulin analogs, excluding two trials with trial-specific dosing/titration algorithms^a^. |
| --- |
| 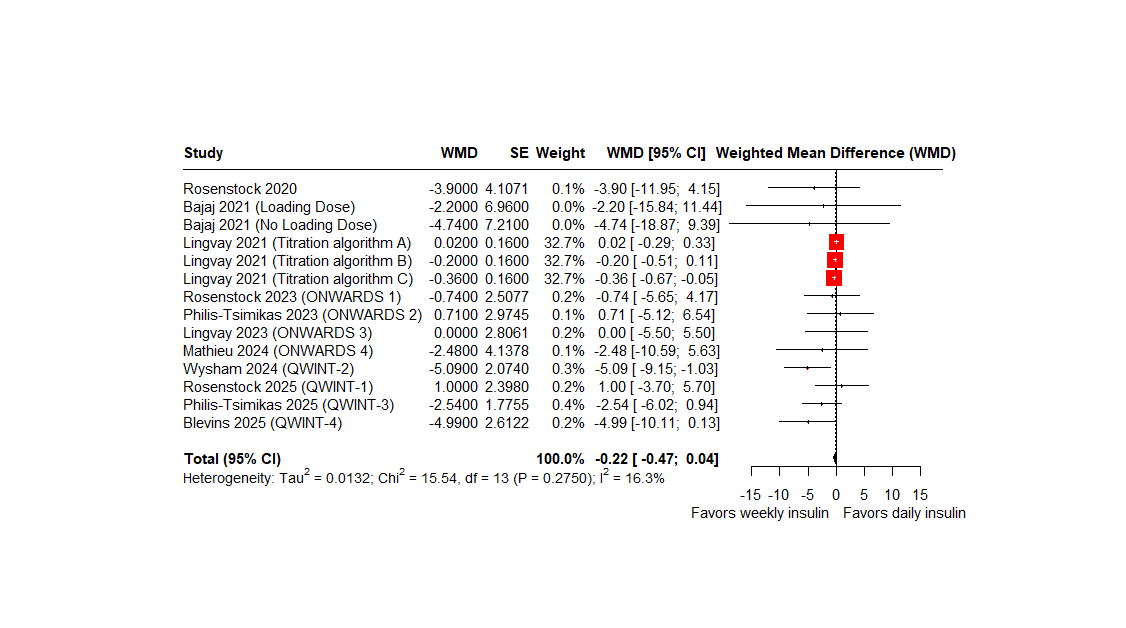 |
| ^a^Studies Bue Valleskey 2023 and Frias 2023 were excluded. |

| **Fig. S11** Difference in mean change in weight (kg) between once-weekly and daily basal insulin analogs. |
| --- |
| 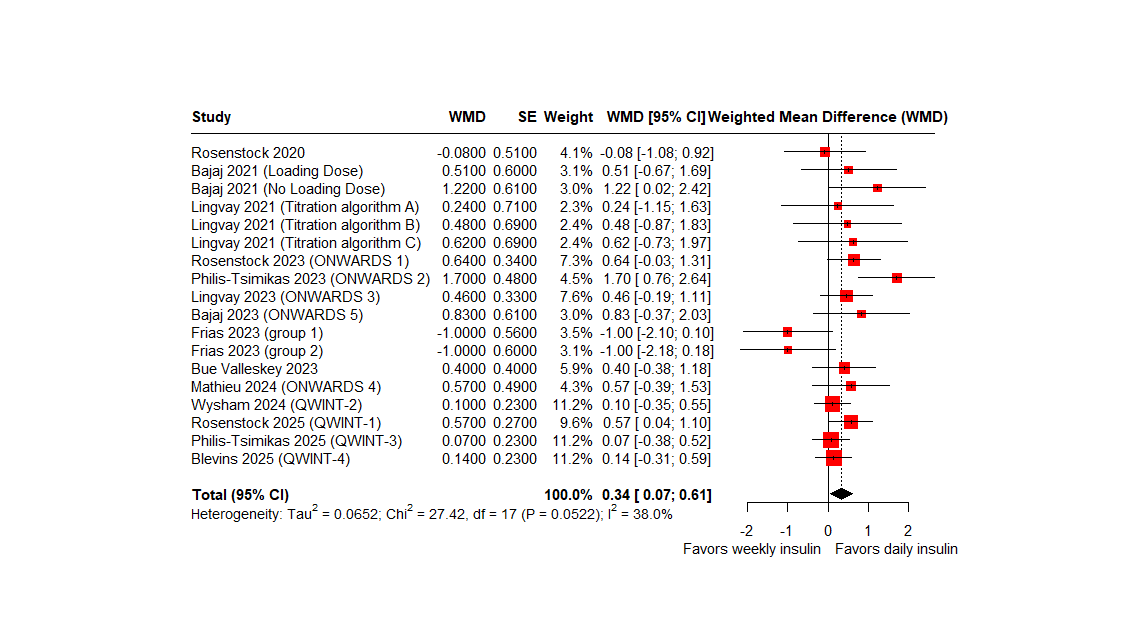 |

| **Fig. S12** Odds ratio of developing level 2 or 3 hypoglycemia between once-weekly and daily basal insulin analogs. |
| --- |
| 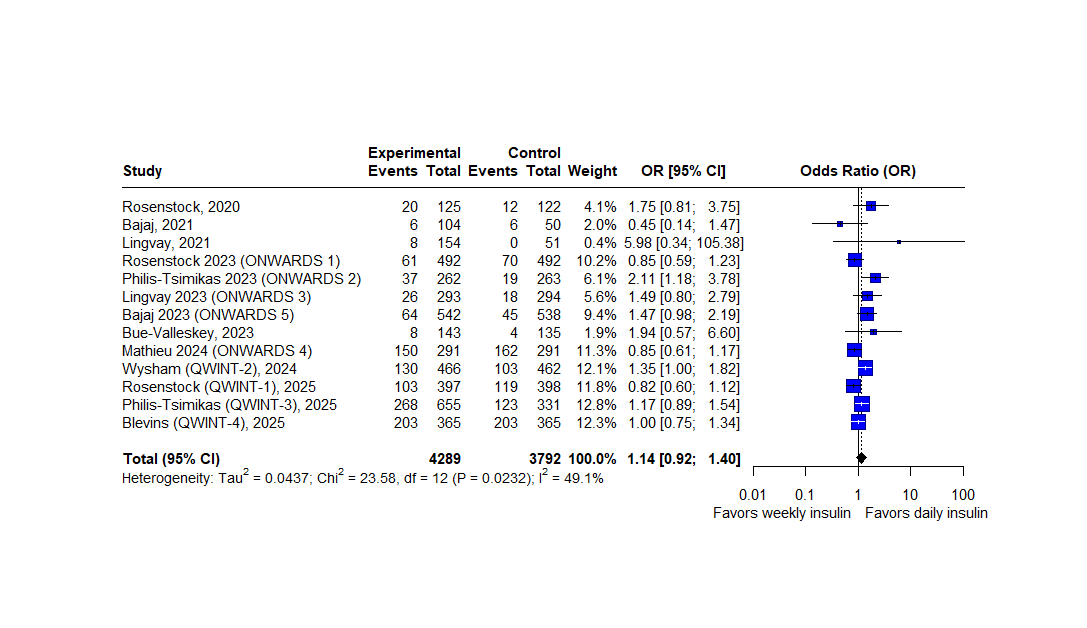 |

| **Fig. S13** Funnel plot assessing small study effects (publication bias). |
| --- |
| 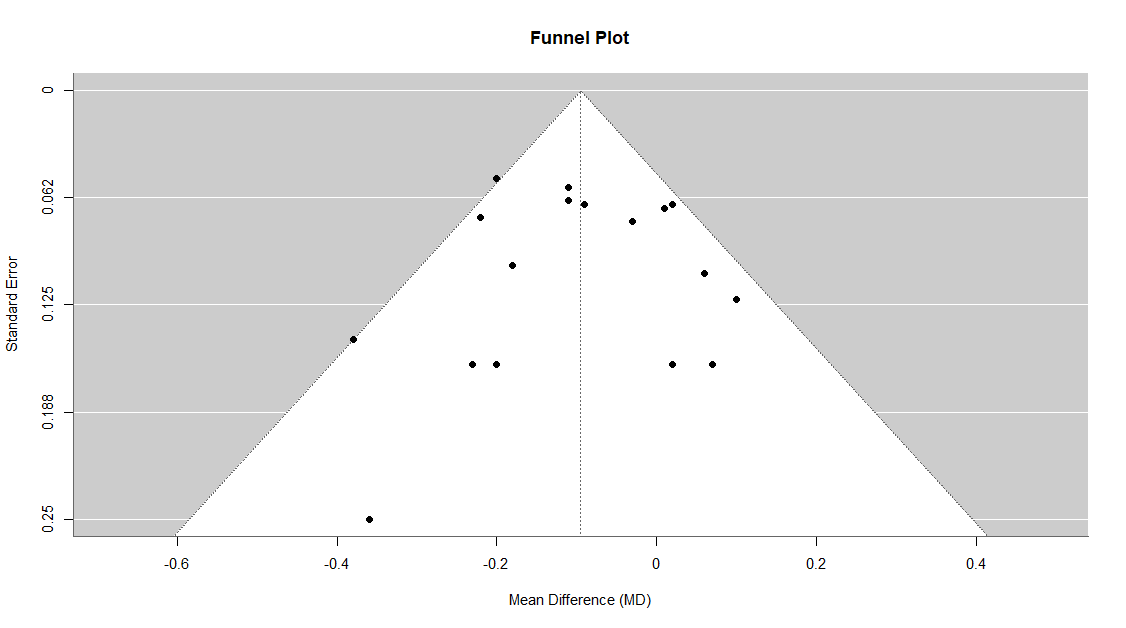 |

## **Table S1**

## MEDLINE/PubMed Search Strategy, as of 05 Jun 2025

| (icodec) OR (BIF) OR (insulin BIF) OR (insulin Fc) OR (basal insulin Fc) OR (LY3209590) OR (insulin efsitora) OR (insulin efsitora alfa) OR (efsitora alfa) OR (efsitora) OR (weekly insulin) OR (once-weekly insulin) OR (once weekly insulin) OR (long acting insulin) OR (long-acting insulin) |
| --- |
| **AND** |
| (type 2 diabetes mellitus) OR (type II diabetes mellitus) OR (T2DM) OR (TIIDM) OR (T2 DM) OR (TII DM) |
| **AND** |
| ((clinical trial) OR (RCT) OR (randomized controlled trial)) |
| **Results**: **1,721** |

## **Table S2**

## Cochrane Library Search Strategy, as of 05 Jun 2025

| ID | **Search** | **Number** |
| --- | --- | --- |
| #1 | MeSH descriptor: [Randomized Controlled Trial] explode all trees | 37 |
| #2 | (randomized controlled trial) OR (randomized control trial) OR (randomized trial) OR (RCT) | 1124455 |
| #3 | MeSH descriptor: [Insulin, Long-Acting] explode all trees | 2440 |
| #4 | (weekly-insulin) OR (weekly insulin) OR (weekly basal insulin) OR (icodec) OR (insulin Fc) OR (BIF) OR (efsitora) OR (efsitora alfa) OR (LY3209590) | 3801 |
| #5 | MeSH descriptor: [Diabetes Mellitus, Type 2] explode all trees | 26718 |
| #6 | (T2DM) OR (TIIDM) OR (T2 DM) OR (TII DM) OR (type 2 diabetes) OR (type II diabetes) | 96804 |
| #7 | #1 OR #2 | 1124455 |
| #8 | #3 OR #4 | 6079 |
| #9 | #5 OR #6 | 96805 |
| #10 | #7 AND #8 AND #9 | **2,253** |

| **Table S3**Clinical Trials database Library Search Strategy, as of 05 June 2025 |
| --- |
| ICODEC OR (insulin 287) OR BIF OR efsitora OR LY3209590 OR (insulin Fc) OR (NNC0148-0287 C) |
| **Results: 125** |

| **Table S4****RoB 2 assessment of the included studies** | | | | | | | |
| --- | --- | --- | --- | --- | --- | --- | --- |
| **Study** | Year | **Randomization process** | **Assignment to intervention** | **Missing outcome data** | **Outcome Measurement** | **Result reporting** | **Overall Risk of Bias** |
| Rosenstock | 2020 | Low | Low | Low | Low | Low | Low |
| Bajaj | 2021 | Low | Low | Low | Low | Low | Low |
| Lingvay | 2021 | Low | Low | Low | Low | Low | Low |
| Rosenstock | 2023 | Low | Low | Low | Low | Low | Low |
| Philis-Tsimikas | 2023 | Low | Low | Low | Low | Low | Low |
| Lingvay | 2023 | Low | Low | Low | Low | Low | Low |
| Mathieu | 2023 | Low | Low | Low | Low | Low | Low |
| Bajaj | 2023 | Low | Low | Low | Low | Low | Low |
| Frias | 2023 | Low | Low | Low | Low | Low | Low |
| Bue-Valleskey | 2023 | Low | High | Low | Low | Low | High |
| Wysham, QWINT-2 | 2023 | Low | Low | Low | Low | Low | Low |
| Rosenstock, QWINT-1 | 2025 | Low | Low | Low | Low | Low | Low |
| Philis-Tsimikas, QWINT-3 | 2025 | Low | Low | Some concerns | Low | Low | Some concerns |
| Blevins, QWINT-4 | 2025 | Low | Low | Low risk | Low | Low | Low |
| **Overall risk of bias** | | | | | | | Low |
